# Inoculating plant growth-promoting bacteria and arbuscular mycorrhiza fungi modulates rhizosphere acid phosphatase and nodulation activities and enhance the productivity of soybean (*Glycine max*)

**DOI:** 10.3389/fpls.2022.934339

**Published:** 2022-09-26

**Authors:** Christopher Ngosong, Blaise Nangsingnyuy Tatah, Marie Noela Enyoe Olougou, Christopher Suh, Raymond Ndip Nkongho, Mercy Abwe Ngone, Denis Tange Achiri, Gylaine Vanissa Tchuisseu Tchakounté, Silke Ruppel

**Affiliations:** ^1^Rhizobiology Group, Department of Agronomic and Applied Molecular Sciences, Faculty of Agriculture and Veterinary Medicine, University of Buea, Buea, Cameroon; ^2^Research Group on Beneficial Microorganisms and Plant Interactions, Leibniz Institute of Vegetable and Ornamental Crops, Großbeeren, Germany; ^3^Institute of Agricultural Research for Development (IRAD), Yaoundé, Cameroon

**Keywords:** fertilizer, N_2_-fixation, P-solubilization, phosphatase, rhizosphere

## Abstract

Soybean [*Glycine max* (L.) Merrill] cultivation is important for its dual role as rich source of dietary protein and soil fertility enhancer, but production is constrained by soil nutrient deficiencies. This is often resolved using chemical fertilizers that exert deleterious effects on the environment when applied in excess. This field study was conducted at Nkolbisson-Yaoundé in the agro-ecological zone V of Cameroon to assess the performance of soybean when inoculated with plant growth-promoting bacteria (PGPB) and arbuscular mycorrhiza fungi (AMF), with or without NPK fertilizer addition. Ten treatments (Control, PGPB, AMF, PGPB+AMF, PGPB+N, PGPB+PK, PGPB+N+PK, PGPB+AMF+N, PGPB+AMF+PK, and PGPB+AMF+N+PK) were established in a randomized complete block design with three replicates. Mycorrhizal colonization was only observed in AMF-inoculated soybean roots. In comparison to control, sole inoculation of PGPB and AMF increased the number of root nodules by 67.2% and 57%, respectively. Co-application of PGPB and AMF increased the number of root nodules by 68.4%, while the addition of NPK fertilizers significantly increased the number of root nodules by 66.9–68.6% compared to control. Acid phosphatase activity in soybean rhizosphere ranged from 46.1 to 85.1 mg h^–1^ kg^–1^ and differed significantly across treatments (*p* < 0.001). When compared to control, PGPB or AMF or their co-inoculation, and the addition of NPK fertilizers increased the acid phosphatase activity by 45.8%, 27%, 37.6%, and 26.2–37.2%, respectively. Sole inoculation of PGPB or AMF and their integration with NPK fertilizer increased soybean yield and grain contents (e.g., carbohydrate, protein, zinc, and iron) compared to the control (*p* < 0.001). Soil phosphorus correlated significantly (*p* < 0.05) with soybean grain protein (*r* = 0.46) and carbohydrate (*r* = 0.41) contents. The effective root nodules correlated significantly (*p* < 0.001) with acid phosphatase (*r* = 0.67) and soybean yield (*r* = 0.66). Acid phosphatase correlated significantly (*p* < 0.001) with soybean grain yield (*r* = 0.63) and carbohydrate (*r* = 0.61) content. Effective root nodules correlated significantly with carbohydrate (*r* = 0.87, *p* < 0.001), protein (*r* = 0.46, *p* < 0.01), zinc (*r* = 0.59, *p* < 0.001), and iron (*r* = 0.77, *p* < 0.01) contents in soybean grains. Overall, these findings indicate strong relationships between farm management practices, microbial activities in the rhizosphere, and soybean performance.

## Introduction

Soybean [*Glycine max* (L.) Merrill] plays crucial roles in food and nutrition security due to its high nutrient contents, while its ability to biologically fix atmospheric nitrogen in symbiosis with Rhizobia enhances the productivity of agricultural systems. Soybean production in Cameroon has been increasing since 2010, and it is the second most cultivated legume after peanuts, with the rapid development of cultivated areas from 6,705 ha in 2008 to 15,020 ha in 2018 (WWF, 2014; Nyahnone, 2017; Nzossié and Bring, 2020). Macroeconomic data show that Cameroon imports an average of 20,000 tons of soybeans worth approximately CFAF 10 billion a year (WWF, 2014), and GMO soybean meal worth CFAF 14 billion (Nyahnone, 2017). Hence, there is a challenge to increase domestic supply to meet agro-industrial demand, which is indicative of the enthusiasm of farmers for soybean production. Wendt and Atemkeng (2004) reported soybean yield ranging between 448 and 709 kg/ha across the first and second planting seasons, with a significant effect of soil nutrients (especially magnesium content) on soybean yield.

Poor soil fertility is a major constraint for crop production in Cameroon with nitrogen (N) and phosphorus (P) as the main limiting elements (Tening et al., 2013; Ngosong et al., 2019; Nanganoa et al., 2020). Soil nutrient deficiencies are commonly resolved using chemical NPK fertilizers that are deleterious to the environment and humans when applied in excess, which has necessitated alternative management practices that can foster crop productivity without jeopardizing sustainability (Ntambo et al., 2017; Mahmud et al., 2020; Mndzebele et al., 2020). A promising alternative to increase crop performance is the use of beneficial microbes to enhance soil fertility, plant nutrition, and protection (Korir et al., 2017; Tchakounté et al., 2020). Despite the widely demonstrated importance of soil beneficial microbes in fostering biotic interactions in the rhizosphere and improving crop performance, biofertilizers have not been fully incorporated in farming systems relative to chemical fertilizers. Although proficient microbes can sustainably improve plant nutrition and protection (Bender et al., 2016; Venneman et al., 2017; Bello et al., 2018), microbial products are still largely untapped in Africa. Hence, there is a need to develop local microbial biofertilizers that can be harnessed to enhance the grain yield and nutrient contents of soybeans (Sogut, 2016; Marro et al., 2020; Zhang et al., 2020).

Besides the high nutrient contents and income generation potential of soybean cultivation, they additionally improve soil nitrogen *via* symbiotic biological N_2_ fixation (Alam et al., 2015; Kalayu, 2019; Soumare et al., 2020). Indigenous or inoculated plant growth-promoting bacteria (PGPB) can improve crop yield *via* biological N_2_ fixation, solubilization of inorganic phosphate, or production of phytohormones (Backer et al., 2018; Rosenblueth et al., 2018; Tchakounté et al., 2018; Bechtaoui et al., 2020). Some microbes mediate crop growth *via* secretion of metabolites, drought tolerance, and protection against pests and diseases (Radhakrishnan et al., 2017). Phosphate-solubilizing bacteria can convert inorganic or organically bound phosphate into bioavailable hydrogen-phosphate ions (H_2_PO_4_^–^ or HPO_4_^2–^) through solubilization and mineralization processes (Behera et al., 2017). These microbes facilitate the conversion of complex forms of N and P to simple available forms for root uptake to enhance crop growth and yield (Kang et al., 2014, 2015; Kuan et al., 2016). Some *Bacillus* spp. release ammonia from nitrogenous organic matter in the soil or have *nifH* gene that produces nitrogenase for N_2_ fixation to supply plants and enhance yield (Ding et al., 2005; Hayat et al., 2010; Kuan et al., 2016). Iron chelation by *Bacillus* spp. *via* siderophore production facilitates solubilization of iron from minerals and organic compounds in the rhizosphere by binding Fe^3+^ in complex substances and reducing them to Fe^2+^ for plant uptake (Walker and Connolly, 2008; Nadeem et al., 2012).

The bacteria biofertilizer used in this study comprised a consortium of symbiotic *Rhizobium* for N_2_ fixation *via* root nodules to directly support the plants, and non-symbiotic PGPB to freely fix N_2_ in the rhizosphere for uptake by soybean roots. In addition, inoculation of arbuscular mycorrhiza fungi (AMF) was intended to boost soybean performance by indirectly modulating soil enzyme activities associated with the processes of N_2_ fixation, P solubilization, and mineralization, or by directly supplying the plants with N and P *via* its hyphal transport network. Arbuscular mycorrhiza fungi (AMF) exert significant positive effects on N_2_ fixation *via* direct or indirect interactions with PGPB through nutrient transport and crop protection (Daniel et al., 2020; Novais et al., 2020). Moreover, microbial activities in the rhizosphere and plant performance can be enhanced by incorporating appropriate amounts of chemical NPK fertilizers in combination with microbial inoculants (Islam et al., 2017; Ntambo et al., 2017; Herliana et al., 2019). Chemical NPK fertilizers were applied in combination with PGPB and/or AMF to assess the possibility of boosting the potential of biofertilizers to enhance soybean performance within the nexus of integrated soil fertility management (Vanlauwe et al., 2010; Kanomanyanga et al., 2021). Hence, this study aims at evaluating microbial dynamics in the soybean rhizosphere, and soybean productivity as influenced by the application of locally produced biofertilizer, with or without the addition of chemical NPK fertilizers. It was hypothesized that inoculating PGPB and AMF will enhance microbial activities in the rhizosphere, including root nodulation and acid phosphatase, and increase the soybean grain yield and nutrient contents.

## Materials and methods

### Experimental site and setup

The experiment was conducted from April to July 2021 at the Institute of Agricultural Research for Development (IRAD) Nkolbisson, Yaoundé, Cameroon. The site is located in agro-ecological zone V of Cameroon, which is a humid forest zone situated between Latitude 03° 8’ 71.2” N and Longitude 11° 45’ 38.0” E. The area has an equatorial climate with a mean annual temperature of 23.5°C (ranging between 16°C and 31°C), and 1,600 mm rainfall that occurs in a bimodal configuration such that the first and second cropping seasons are separated by a 4-month dry season, which lasts from mid-March to early July and from late August to mid-November, respectively (Ambassa-Kiki and Nill, 1999). However, the duration of each season presently varies and the second season rains are erratic due to global climate change dynamics.

Rice was previously cultivated on the field site from 2016 to 2019 and fallowed for 1 year in 2020 before this study. The field experiment was laid out as a randomized complete block design with ten treatments and three replicates each, giving a total of thirty experimental units. The treatments include T1 – control (no input), T2 – plant growth-promoting bacteria (PGPB), T3 – Arbuscular mycorrhiza fungi (AMF), T4 – PGPB+AMF, T5 – PGPB+N, T6 – PGPB+PK, T7 – PGPB+N+PK, T8 – PGPB+AMF+N, T9 – PGPB+AMF+PK, and T10 – PGPB+AMF+N+PK. Each experimental plot measured 3.2 m × 4 m (12.8 m^2^) with a 1 m buffer zone between plots and a 1.5 m buffer between the replicate blocks.

**FIGURE 1:**
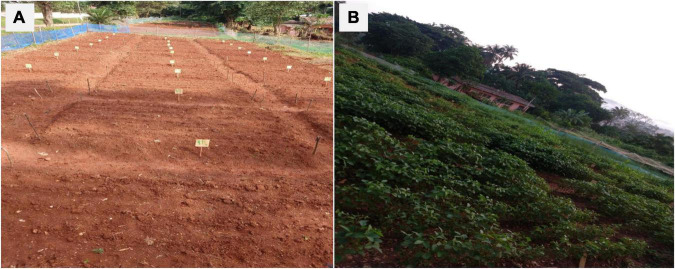
Experimental site with tilled soil beds before sowing **(A)** and soybean plants at full podding **(B)**.

The experimental site was cleared and weed regrowth was allowed for 2 weeks, and the emerging weeds were sprayed with a systemic herbicide (Roundup 360SL, Belgium; comprising active components glyphosate) at a rate of 129.6 kg in 360 L of water ha^–1^. Three days after the application of herbicide, all plots were tilled manually to produce raised beds of approximately 30 cm high (Figure 1), and soybean was planted 1 week after herbicide spray. Three soybean seeds (Panorama 357 variety) were planted per hole at approximately 4 cm depth and 10 cm intra-row and 40 cm inter-row spacing, making 7 rows per plot. Thinning was done after germination to two vigorous plants per hole, giving a total of 382,813 plants ha^–1^. Synthetic insecticide K-Optimal (SCPA SIVEX International France; comprising active components Lambda—cyhalothrine 15 g L^–1^ + Acetamipride 20 g L^–1^) and fungicide Monchamp 72 WP (Mancozeb 60% + Metalaxyl 12%) were applied at 3 weeks after sowing to control insect pests and fungal infections. Each was applied at the rate of 150 ml in 15 L of knapsack sprayer, which is equivalent to 2 L ha^–1^ each. The field was regularly monitored and weeding was done manually.

### Microbial inoculation

#### Plant growth-promoting bacteria

The bacterial inoculant used in this study consisted of a consortium of symbiotic and non-symbiotic plant growth-promoting bacteria—PGPB (Table 1). The non-symbiotic PGPB included the following organisms: (03) *Arthobacter* sp., (03) *Bacillus* sp., (01) *Lysinibacillus* sp., (03) *Paenibacillus* sp., and (01) *Sinomonas* sp., which were isolated from the rhizosphere of maize plants in Cameroon (Tchakounté et al., 2018); and (01) *Kosakosania radicincitans* isolated from the phyllosphere of winter wheat in Germany and deposited in NCBI as DSM 16656*^T^* GenBank: CP018016.1, CP018017.1, CP018018.1 (Ruppel and Merbach, 1995; Becker et al., 2018). The symbiotic bacterium (*Bradyrhizobium japonicum*) was obtained from the Soil Microbiology Laboratory of the Biotechnology Center of the University of Yaoundé I, Cameroon.

**TABLE 1:** Plant growth-promoting bacteria (PGPB) and arbuscular mycorrhiza fungi, their functional traits, and potential roles in the consortium of biofertilizers to enhance soil fertility and performance of soybean plants.

S/N	Beneficial microbes		Microbial isolates	Genus	Family	Phylum	nifH gene	Siderophore production	Phosphorus solubilization	Biocontrol activity	Reason for selection and inclusion in the inoculant consortium
1	PGPB	Symbiotic	NKa11	*Bradyrhizobium*	Rhizobiaceae	Proteobacteria	√	/	/	/	N_2_ fixation
2			*DSM 16656^T^	*Kosakonia*	Enterobacteriaceae	Proteobacteria	√	√	√	√	N_2_ fixation, Siderophore production and P solubilization
3		Non-symbiotic	V64 (*MN128891), V84 (*MN128892), and V127	*Arthrobacter*	Micrococcaceae	Actinobacteria	/	√	√	/	Siderophore production and P solubilization
4			VA9, V22, and V65	*Bacillus*	Bacillaceae	Firmicutes	/	√	√	/	Siderophore production and P solubilization
5			V47	*Lysinibacillus*	Bacillaceae	Firmicutes	√	/	/	/	N-Fixation
6			VA7	*Paenibacillus*	Paenibacillaceae	Firmicutes	/	√	√	/	P solubilization and N_2_ Fixation
7			V12 and V18	*Paenibacillus*	Paenibacillaceae	Firmicutes	√	/	√	/	P solubilization and N_2_ Fixation
8			V4	*Sinomonas*	Micrococcaceae	Actinobacteria	/	√	√	/	Siderophore production and P solubilization
9	Endophytic fungi		*Enthophospora infrequens*, *Scutellospora cerradessis*, and *Gigaspora gigantea*	Mycorrhiza	Acaulosporaceae -Gigasporaceae	Glomeromycota	/	/	√	√	P solubilization and mobilization

*NCBI accession reference.

For the production of microbial inoculant consortium, a colony of symbiotic *B. japonicum* was collected from an inoculum stock and transferred into a 500-ml flask containing 100 ml sterilized yeast mannitol broth (YMB), and incubated in a shaker at 28°C at 200 rpm for 48 h. From a stock culture of each of the non-symbiotic plant growth-promoting bacteria, a pure colony was collected and transferred into a 500-ml flask containing 100 ml sterilized nutrient broth (Standard nutrient broth I, Carl Roth, Germany), and incubated at 28°C for 24–48 h. The individually cultured symbiotic and non-symbiotic plant growth-promoting bacteria were assembled into a microbial consortium in a 5 L container and sugar was added (1:1) to serve as an adjuvant because the *B. japonicum* used in this study is not sticky as compared to the non-symbiotic microbes. The soybean seeds were immersed in the microbial inoculant consortium (e.g., 1 kg of soybean seeds per 100 ml of biofertilizer inoculum) and thoroughly mixed. The seeds were removed from the inoculum and allowed to air-dry for 1 h before planting (Atieno et al., 2012).

#### Arbuscular mycorrhiza fungi

The composite inoculant product used for this experiment was obtained from the Regional Biocontrol and Applied Microbiology Laboratory of IRAD, Nkolbisson, Cameroon. It comprised the three most dominant arbuscular mycorrhiza strains (*Enthophospora infrequens*, *Scutellospora cerradessis*, and *Gigaspora gigantea*) identified in the soybean rhizosphere of six high-intensity soybean-producing areas across the five different agro-ecological zones of Cameroon. In total, 300 g of mycorrhiza inoculum stock was used to produce a bulk inoculum using a sterilized mixture of fine and coarse sand substrates (1:1), sterilized at 121°C for 1 h in an autoclave (model PTS-B100L). Three polypropylene bags were filled with 20 kg each of sterilized substrates, and highly mycotrophic *Sorghum bicolor* plants were planted and maintained in the greenhouse for 3 months with regular irrigation, before subjecting the plants to water stress for 1 month to stimulate sporulation by mycorrhiza. The suitability of the mycorrhiza inoculum was determined by the spore density, which was determined from a 100 g sample of the homogenized inoculant product by immersing in 300 ml distilled water in a 1,000-ml beaker, and the mixture was stirred and allowed to stand for 15 s. Four sieves were arranged in decreasing order of mesh size (710, 225, 125, and 45 μm) and used to filter the supernatant of each mixture. The contents of the last three sieves were collected, washed, and the number of observed mycorrhiza spores was counted on a square graduated petri-dish using a stereo microscope (WILD M2B, Germany). The observed spore density of 670 spores 100 g^–1^ of inoculum was considered suitable for use as AMF inoculum. The AMF inoculum was air-dried and used for field inoculation at 20 g per soybean stand (containing approximately 134 spores). The inoculum was applied by placing the 20 g inoculum at about 80 mm depth in the planting hole before planting soybean seeds.

### Application of chemical fertilizers

So far there is no specific fertilizer recommendation for soybean production on the study site. Therefore, the application of nitrogen as urea, phosphorus as triple superphosphate (TSP), and potassium as muriate of potash (MOP) was based on general fertilizer recommendations for soybean. Urea was applied at the rate of 40 kg N ha^–1^ (Uko et al., 2002) as two split doses of 20 kg N ha^–1^ each 2 weeks after planting and beginning of seed development at the R5 stage. Phosphorus was applied at 30 kg P_2_O_5_ ha^–1^ (Lamptey et al., 2014) and potassium at 40 kg K_2_O ha^–1^ (Islam et al., 2017) 2 weeks after planting. All fertilizers were applied by ringing at approximately 5 cm from plants to avoid burns and minimize nutrient loss through leaching and volatilization.

### Data collection

#### Soil properties

Pre-planting soil was sampled for the entire experimental site after clearing and laying out but before tillage, while post-planting soil was sampled for each plot at harvest. An auger was used to collect three pre-planting soil samples randomly using a Z-pattern at 0–15 cm depth and bulked to form a composite sample. Three post-planting soil samples were also collected randomly from each treatment plot at 0–15 cm depth using an auger, and thoroughly mixed to form a composite sample. All soil samples were air-dried at room temperature and stored in polybags before analysis. The soil samples were crushed and sieved through a 2-mm sieve for the determination of soil’s physical and chemical properties.

The soil particle size distribution was determined using the pipette sampling method with sodium hexametaphosphate as a dispersing agent, and the textural class was assigned according to USDA textural triangle (Van Reeuwijk, 1992). The soil pH was determined potentiometrically in water (H_2_O) and 1 N potassium chloride (KCl) solutions after 24 h in soil suspension (soil/liquid 1:2.5 w/v) using a glass electrode pH meter. The exchangeable bases (Ca^2+^, Mg^2+^, K^+^, and Na^+^) were extracted using 1 N ammonium acetate (NH_4_CH_3_CO_2_) solution at pH 7. Calcium (Ca) and magnesium (Mg) were determined by the titration method using Eriochrome Black T (EBT or Erio T) as an indicator while potassium (K) and sodium (Na) were determined using the flame photometer (Rowell, 1994). Exchange acidity was extracted with 1 N KCl and determined by titrating the extract with 0.01 N NaOH, using a phenolphthalein indicator (Van Reeuwijk, 1992). Effective cation exchange capacity (ECEC) was determined by the summation of exchangeable bases and exchange acidity. The total soil nitrogen (N) was determined by the macro Kjeldahl digestion method (Bremner and Mulvaney, 1982). Plant available phosphorus (P) in the soil was determined by the Bray II method (Van Reeuwijk, 1992), and organic carbon was determined by the wet oxidation method (Kalra and Maynard, 1991).

#### Mycorrhiza colonization

**FIGURE 2:**
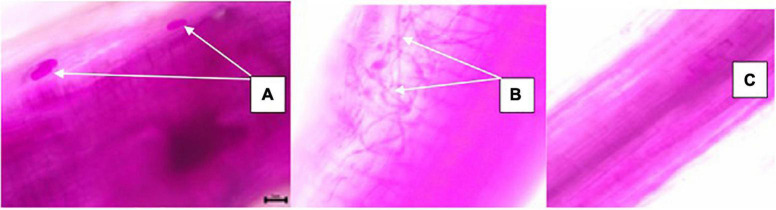
Microscopic identification of mycorrhiza structures in soybean roots; Vesicles **(A)**, Hyphae **(B)**, and un-infected roots **(C)** as influenced by inoculation of plant growth-promoting bacteria (PGPB) and arbuscular mycorrhiza fungi (AMF), with or without the addition of nitrogen (N), phosphorus (P), and potassium (K) fertilizers.

An assessment of AMF root colonization was conducted according to Begoude et al. (2016). Briefly, 1–2 cm segments of root samples were placed in 5% KOH solution for 24 h at room temperature and rinsed three times with water on a fine sieve. Root samples were acidified in 10% HCl (v/v) for 15 min and stained with 0.01% (w/v) fuchsine acid for 24 h at room temperature. Root segments were randomly selected from the stained samples and three replicates of 10 roots per slide were assessed for the occurrence of AMF structures (e.g., vesicles, arbuscules, and hyphae) using an optical microscope (Biological compound microscope with replaceable LED light, OMAX 40X-2500X Trinocular, Germany) (Figure 2). The mycorrhizal frequency (F%) was given as the ratio of colonized root fragments to the total number of observed root fragments.

#### Quantification of rhizosphere acid phosphatase activity

Five plants were randomly selected from the center of each plot at full podding for assessment of acid phosphatase activity. A spade was used to dig approximately 5 cm around each plant at the depth of 20 cm, and 1 g root adhering soil was collected from each plant and bulked to form a composite sample of 5 g, from which 1 g was taken into a microcentrifuge tube and 0.5 ml of 100 mM phosphate buffer was added. P-nitrophenyl phosphate (p-NPP, 10 mM) in 100 μl solution was used as substrate. The final volume of the reaction mixture was adjusted to 1 ml by adding the required amount of distilled water. The tube was vortexed (2 min) at room temperature and incubated at 37°C for 60 min in shake condition (100 rpm). After the incubation, samples were centrifuged at 10,000 rpm (5 min) and the clear supernatants were transferred into clean test tubes and 2 ml of 1 M NaOH was added. The yellow filtrate was analyzed using a colorimeter (Klett colorimeter, Clinical model, 800-3, 115 VAC) at λ = 430 nm. The concentration of soluble protein in the supernatant was determined using the Bradford reactive procedure, and the specific activity of soil acid phosphatase was estimated using the formula:


(1) $$\mathrm{Protein}\,\mathrm{content}=\frac{10\times C}{17800}$$

Where, 10 = constant, C = concentration of soluble protein, and 17,800 is the molecular extension coefficient of phosphatase. All derived values were then converted to mg h^–1^ kg^–1^ soil by multiplying by 100,000.

#### Root nodulation

Five plants were randomly selected from the second border rows of each plot at full podding and assessed for nodulation parameters. The nodulation rating was assessed according to Tamiru et al. (2012) as follows:


(2) Nodulation⁢rating=

$$\frac{\left(a\times 10\right)+\left(b\times 5\right)+\left(c\times 1\right)+\left(d\times 0\right)}{\mathrm{Total}\,\mathrm{number}\,\mathrm{of}\,\mathrm{plants}}$$

Where the number of plants showing tap root nodulation (a), plants with nodules in secondary roots but close to tap root (b), plants with scattered nodulation (c), and plants without nodulation (d) were evaluated.

The nodule volume was also assessed according to Tamiru et al. (2012), where nodules of the five sampled plants were immersed in a 50 ml plastic cylinder containing 30 ml of water. The amount of water displaced after immersion of the nodules was recorded and the mean was considered as nodule volume per plant. For the effectiveness of root nodules, all the nodules from the five sampled plants per treatment were dissected using a sharp knife, and a hand lens was used to observe the internal nodule color. The presence of pink or reddish coloration was considered as effective and used to distinguish nodules that are actively fixing nitrogen from the inactive nodules (Ngeno et al., 2012).

#### Soybean grain yield

Soybean plants were harvested at physiological maturity and the weight of 1,000 randomly selected grains from each plot maintained at 10% moisture content was reported as a thousand grain weight. Soybean grain yield was obtained by adjusting the moisture level to 10% according to the following formula and converted to tons ha^–1^:


(3) $$A\,d\,j\,u\,s\,t\,e\,d\,y\,i\,e\,l\,d=\frac{100-M\,C}{100-10}\times u\,n\,a\,d\,j\,u\,s\,t\,e\,d\,y\,i\,e\,l\,d$$

Where MC is the moisture content of soybean seeds at the time of measurement, and 10 is the percentage standard moisture content of soybean seeds at harvest.

#### Nutrient contents of soybean grains

The proximate (e.g., protein and carbohydrate) composition of soybean grains were determined according to the official method described by the Association of Official and Analytical Chemist (AOAC, 2005). Each sample was analyzed in triplicates and values were presented in percentages. The Anthrone standard method (David, 1978) was used to estimate the content of carbohydrates where different volumes of glucose solution from 200 μg mol^–1^ stock solution were pipetted and made up to 1 ml with distilled water. In total, 15 tubes were used with tube 1 considered as blank, tubes 2–9 were used to construct a standard curve, and tubes 10–15 were used for unknown samples. Anthrone (5 ml) was added to each tube and vortexed to thoroughly mix and allowed to cool. The tubes were covered with marble/caps and incubated at 90°C for 17 min and allowed to cool at room temperature, and optical density was measured at 620 nm against the blank sample. The amount of glucose in the unknown sample was determined by plotting a standard curve of A620 on Y-axis and μg glucose on X-axis.

Crude protein was determined using the Kjeldahl digestion method by measuring the nitrogen content of the soybean grain samples and multiplying it by a factor of 6.25, based on the fact that protein contains approximately 16% nitrogen. Approximately 2 g of crushed soybean grain powder was weighed into a Kjeldahl flask and 25 ml of concentrated sulfuric acid, 0.5 g of copper sulfate, 5 g of sodium sulfate, and a speck of selenium tablet acid were added. Heat was first applied in a fume cupboard slowly to prevent undue frothing. The digestion continued for 45 min until the digestate became clear pale green. The digestate was transferred into a 100 ml volumetric flask and this was made up to the mark with distilled water. The Kjeldahl distillation apparatus used for distillation was steamed up and 10 ml of the digest was added into the apparatus *via* a funnel and allowed to boil. Sodium hydroxide (10 ml) was added from the measuring cylinder so that ammonia was not lost. It was later distilled into 50 ml of 2% boric acid containing screened methyl red indicator. The contents of the collecting flask (50 ml) were titrated with sulfuric acid standard volumetric solution using a burette and the amount of titrant used was read. When colorimetric end-point detection was applied, the end-point was reached when the color of the solution changed from green to red. The burette reading was estimated to the nearest 0.01 ml. To confirm that the reagents were free from nitrogen, a blank test was conducted (e.g., performing digestion, distillation, and titration) using only reagents without adding soybean material. The nitrogen content of soybean grains was calculated as follows:


(4) $$\text{MN}=\frac{\left(V\,a-V\,b\right)\times C\,H\,C\,l\times M\,N}{m\,v\,z}\times 1,000$$

where Va = volume of standard HCl solution when titrating sample, Vb = volume of standard HCl solution when titrating blank, CHCl = concentration of HCl (mol L^–1^), MN = nitrogen molar mass (g mol^–1^), and mvz = weight of sample (g).


(5) Percentagecrudeprotein=%Nitrogenx 6.25

The mineral content of zinc (Zn) and iron (Fe) were determined using atomic absorption spectrometry according to the standard method (AOAC, 2005). Samples were ashed at 550°C and boiled with 10 ml of 20% hydrochloric acid in a beaker and then filtered into a 100 ml standard flask. This was made up to the mark with distilled water. The mineral content of Zn and Fe was determined from the resulting solution using Atomic Absorption Spectrophotometer at 510, 213.86, 766.5, 213.6 nm. Different electrode lamps were used for each mineral and the equipment was run for standard solutions of each mineral before and during determination to ascertain the efficiency. All values were expressed in mg 100 g^–1^.


(6) Z⁢n/F⁢e⁢(m⁢g/100⁢g)=

$$\frac{A\,b\,s\,o\,r\,b\,e\,n\,c\,y\,\left(p\,p\,m\right)\times d\,i\,l\,u\,t\,i\,o\,n\,f\,a\,c\,t\,o\,r\times v\,o\,l\,u\,m\,e\,o\,f\,e\,x\,t\,r\,a\,c\,t\,\left(m\,L\right)}{W\,e\,i\,g\,h\,t\,o\,f\,s\,a\,m\,p\,l\,e}$$

### Data analysis

All statistical analyses were done using SPSS (Ver. 23), and data sets were analyzed for normality and homogeneity using Kolmogorov–Smirnov and Levene’s tests, respectively. Data on soil chemical properties, root nodulation, acid phosphatase activity, grain yield, and nutrient contents were subjected to analysis of variance (ANOVA, *p* < 0.05) to test the effects of treatments as categorical predictors. Significantly different means were separated using Tukey’s HSD test (Tukey’s HSD, *p* < 0.05). Where applicable, correlation (*p* < 0.05) was performed to determine the degree of association between the dependent and independent variables.

## Results

### Soil chemical properties

The baseline soil analysis before establishing the experiment indicates an acidic soil with very low soil nutrient contents (e.g., N, P, Ca, Mg, K, Na) and cation exchange capacity (Table 2). Only the organic C content (1.4–2.4%) is suitable, which results in a quite wide pre-experiment C/N ratio of approximately 20 units. The post-planting soil Ca and Mg contents differed significantly across the experimental treatments. When compared to pre-planting soil, treatments with or without microbial and chemical fertilizer application increased the soil calcium content significantly (F*_9,20_* = 3.78, *p* < 0.05, Table 2), which ranged from 2.40 to 7.26 cmol kg^–1^, with the highest in the sole mycorrhiza treatment and the lowest in PGPB+AMF+NPK. The post-planting magnesium content ranged from 1.60 to 8.30 cmol kg^–1^ of soil, and it was significantly higher in the PGPB+AMF+NPK treatment (F*_9,20_* = 3.43, *p* < 0.05, Table 2).

**TABLE 2:** Soil properties as influenced by inoculation of plant growth-promoting bacteria (PGPB) and arbuscular mycorrhiza fungi (AMF), with or without the addition of nitrogen (N), phosphorus (P), and potassium (K) fertilizers.

Treatments	pH (H_2_O)	pH (KCl)	Total N	Organic C	Organic M	C/N	Bray P	Ca	Mg	K	Na	ECEC
			_______________(%)_______________				(mg kg^–1^)	____________________cmol kg^–1^____________________				
Pre-plant	4.30 ± 0.17a	3.23 ± 0.06ab	0.09 ± 0.02a	2.16 ± 0.27a	3.73 ± 0.47a	20.66 ± 2.93a	5.56 ± 1.02a	2.40 ± 0.13d	4.13 ± 1.62b	0.76 ± 0.12a	0.03 ± 0.00b	17.70 ± 1.55a
Control	4.37 ± 0.15a	3.10 ± 0.10b	0.10 ± 0.03a	2.36 ± 1.05a	4.06 ± 1.81a	24.16 ± 11.47a	4.23 ± 0.32a	6.51 ± 0.85abc	1.60 ± 0.92b	1.42 ± 1.21a	0.42 ± 0.27ab	22.19 ± 6.75a
PGPB	4.27 ± 0.12a	3.40 ± 0.17ab	0.07 ± 0.02a	2.45 ± 0.59a	4.23 ± 1.01a	41.55 ± 24.04a	4.80 ± 0.31a	5.71 ± 0.53abc	1.76 ± 0.57b	2.73 ± 2.78a	0.14 ± 0.03ab	22.08 ± 6.41a
AMF	4.37 ± 0.23a	3.50 ± 0.10a	0.11 ± 0.03a	2.40 ± 0.83a	4.13 ± 1.43a	23.95 ± 15.35a	5.88 ± 0.64a	7.26 ± 0.83a	2.18 ± 0.81b	2.50 ± 1.39a	0.15 ± 0.09ab	20.48 ± 7.92a
PGPB+AMF	4.43 ± 0.06a	3.20 ± 0.10ab	0.09 ± 0.04a	2.36 ± 0.47a	4.06 ± 0.80a	30.24 ± 17.61a	5.45 ± 0.43a	6.81 ± 1.08ab	1.89 ± 1.45b	1.93 ± 0.76a	0.34 ± 0.10ab	22.61 ± 1.29a
PGPB+N	4.23 ± 0.25a	3.20 ± 0.10ab	0.11 ± 0.02a	2.32 ± 0.47a	3.99 ± 0.61a	21.99 ± 1.93a	5.17 ± 0.86a	4.78 ± 0.37abc	2.48 ± 0.64b	1.59 ± 1.10a	0.23 ± 0.13ab	21.33 ± 0.88a
PGPB+PK	4.27 ± 0.21a	3.33 ± 0.15ab	0.11 ± 0.06a	1.46 ± 0.36a	2.53 ± 0.81a	14.75 ± 5.46a	8.56 ± 0.99a	5.21 ± 0.88abc	3.44 ± 3.45b	1.02 ± 0.39a	0.15 ± 0.15ab	18.56 ± 1.62a
PGPB+NPK	4.23 ± 0.1a	3.30 ± 0.10ab	0.10 ± 0.01a	1.81 ± 0.12a	3.13 ± 0.21a	18.74 ± 1.07a	9.34 ± 0.36a	5.15 ± 0.67abc	1.81 ± 1.01b	0.50 ± 0.30a	0.10 ± 0.11ab	13.23 ± 5.18a
PGPB+AMF+N	4.23 ± 0.3a	3.33 ± 0.15ab	0.05 ± 0.04a	2.12 ± 0.71a	3.66 ± 1.22a	64.28 ± 52.65a	8.95 ± 4.76a	5.79 ± 1.21abc	2.56 ± 1.76b	0.69 ± 0.13a	0.06 ± 0.02b	24.29 ± 13.47a
PGPB+AMF+PK	4.37 ± 0.1a	3.43 ± 0.12ab	0.08 ± 0.02a	1.81 ± 0.53a	3.13 ± 0.91a	23.63 ± 10.69a	7.47 ± 3.49a	4.35 ± 1.01bc	2.86 ± 2.48b	0.56 ± 0.06a	0.03 ± 0.00b	17.72 ± 3.85a
PGPB+AMF+NPK	4.27 ± 0.40a	3.20 ± 0.10ab	0.06 ± 0.02a	2.36 ± 0.32a	4.06 ± 0.56a	40.77 ± 15.13a	8.58 ± 2.00a	4.11 ± 1.40c	8.29 ± 2.80a	0.62 ± 0.24a	0.03 ± 0.03b	16.88 ± 1.47a

Data (Mean ± SD) within columns with different letters are significantly different (Tukey’s HSD, *p* < 0.05).

### Mycorrhiza colonization and nodulation of soybean roots

**FIGURE 3:**
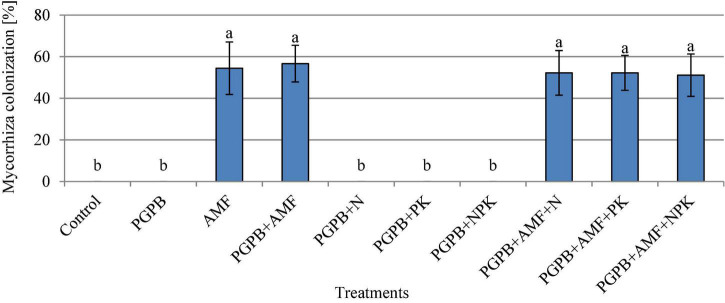
Mycorrhiza colonization of soybean roots affected by inoculation of plant growth-promoting bacteria (PGPB) and arbuscular mycorrhiza fungi (AMF), with or without the addition of nitrogen (N), phosphorus (P), and potassium (K) fertilizers. Data (Mean ± SD) with different letters are significantly different (Tukey’s HSD, *p* < 0.05).

**FIGURE 4:**
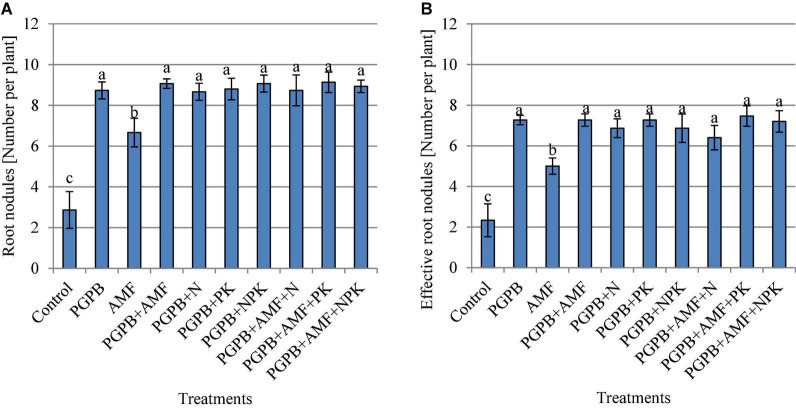
The total number of root nodules per plant **(A)** and the number of effective root nodules per plant **(B)** as affected by inoculation of plant growth-promoting bacteria (PGPB) and arbuscular mycorrhiza fungi (AMF), with or without the addition of nitrogen (N), phosphorus (P), and potassium (K) fertilizers. Data (Mean ± SD) with different letters are significantly different (Tukey’s HSD, *p* < 0.001).

The soybean roots were colonized by mycorrhiza at the rate of 51.1–56.7%, which was only observed in plant roots that were inoculated with AMF, but there was no significant difference between the treated plots (*p* > 0.05, Figure 3). Hence, the addition of PGPB or chemical NPK fertilizer to the AMF treatments did not influence the rate of colonization of soybean roots by AMF. The number of root nodules ranged from 2.9 to 9.1 with the highest in PGPB treatments followed by the sole AMF as compared to the control (F*_9,20_* = 37.53, *p* < 0.001; Figure 4A). The inoculation of PGPB and AMF increased the number of root nodules by 67.2% and 57%, respectively, as compared to the control, with the PGPB performing 10.2% more than AMF in relation to the control. Meanwhile, the integration of PGPB and AMF increased the number of root nodules by 68.4% compared to the control. Furthermore, the treatments with co-application of biofertilizers (PGPB and AMF) and mineral NPK fertilizer significantly increased the number of root nodules at the range of 66.9–68.6% compared to the control, but there was no significant difference between the treated plots (Figure 4A). A similar trend of results was also observed for the number of effective nodules (F*_9,20_* = 28.85, *p* < 0.001; Figure 4B). The highest nodulation rating occurred in PGPB treatments compared to the control (F*_9,20_* = 8.36, *p* < 0.05; Supplementary Table 1). Nodule volume ranged from 0.16 to 0.40 ml per plant and differed significantly (F*_9,20_* = 22.15, *p* < 0.05; Supplementary Table 1) with the highest in PGPB treatments. The nodule dry weight ranged from 0.017 to 0.038 g with the highest in PGPB treatments compared to the control (F*_9,20_* = 43.38, *p* < 0.05; Supplementary Table 1). Overall, no additional effect of mineral NPK fertilizer application was detected in all the measured root nodulation parameters.

### Acid phosphatase activity in the soybean rhizosphere

**FIGURE 5:**
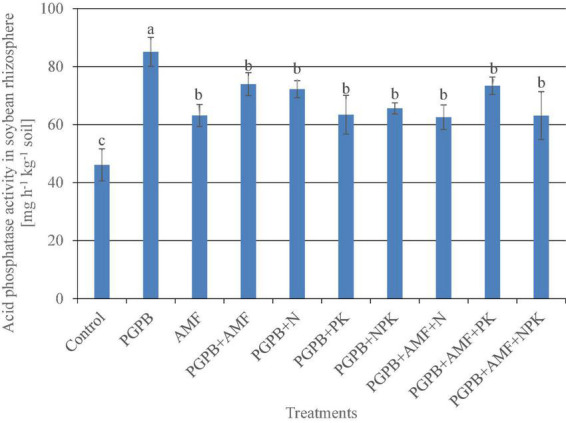
Acid phosphatase activity in the rhizosphere of soybean plants affected by inoculation of plant growth-promoting bacteria (PGPB) and arbuscular mycorrhiza fungi (AMF), with or without the addition of nitrogen (N), phosphorus (P), and potassium (K) fertilizers. Data (Mean ± SD) with different letters are significantly different (Tukey’s HSD, *p* < 0.001).

The acid phosphatase activity in the soybean rhizosphere ranged from 46.1 to 85.1 mg h^–1^ kg^–1^, which differed significantly across treatments (F*_9,20_* = 13.25, *p* < 0.001; Figure 5), with the lowest phosphatase activity recorded in the soybean roots of the control without any microbial or mineral fertilizer application, while the highest occurred in the sole PGPB treatment as compared to all the other treatments. All the microbial treatments almost doubled the acid phosphatase activity in the soybean rhizosphere as compared to the control, with a 45.8% and 27% increase in acid phosphatase activity for PGPB and AMF, respectively, as compared to the control, while the PGPB performed 18.8% more than AMF in relation to the control. Meanwhile, the integration of PGPB and AMF increased the acid phosphatase activity by 37.6% compared to the control, but this was 8.2% lower than the sole PGPB application. Furthermore, the treatments with co-application of biofertilizers (PGPB and AMF) and mineral NPK fertilizer significantly increased the acid phosphatase activity in the range of 26.2–37.2% compared to the control, but there was no significant difference between the NPK fertilizer–treated plots (Figure 5). Meanwhile, the co-application of biofertilizers (PGPB and AMF) and mineral NPK fertilizer significantly reduced the acid phosphatase activity by 13.7–26.5% as compared to the sole PGPB treatment.

### Soybean grain yield and nutrient contents

**FIGURE 6:**
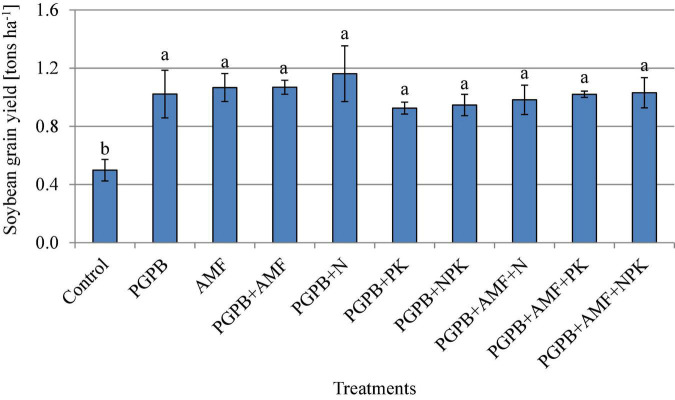
Soybean grain yield (tons ha^1^) affected by inoculation of plant growth-promoting bacteria (PGPB) and arbuscular mycorrhiza fungi (AMF), with or without the addition of nitrogen (N), phosphorus (P), and potassium (K) fertilizers. Data (Mean ± SD) with different letters are significantly different (Tukey’s HSD, *p* < 0.001).

The soybean grain yield ranged between 0.50 and 1.16 tons ha^–1^ and increased significantly in all applied treatments compared to the non-treated control (F*_9,20_* = 8.83, *p* < 0.001; Figure 6). The soybean grain yield increased significantly by 46.1–57.1% for all treated plots (sole PGPB or AMF and their combination with NPK fertilizer) in relation to the control, but there was no significant difference between the treated plots. In contrast to soybean grain yield that was not influenced by the integrated application of biofertilizers (PGPB or AMF) and chemical NPK fertilizers, the nutrient contents of soybean grains increased significantly in the integrated biofertilizer and NPK fertilizer treatments as compared to the sole application of biofertilizers (Table 3). All treatments, including biofertilizers alone and their combinations with chemical NPK fertilizers, significantly increased the contents of carbohydrate, protein, zinc, and iron in the soybean grains (Table 3). The carbohydrate content ranged between 20.1 and 22.7% (F*_9,20_* = 5926.53, *p* < 0.001) and protein ranged between 31 and 39.9% (F*_9,20_* = 3977.2, *p* < 0.001) across treatments, with the highest in PGPB+AMF+NPK treatment, as compared to the lowest values in the control (Table 3). Inoculating PGPB and AMF with or without NPK fertilizers significantly enhanced the protein content of soybean (F*_9,20_* = 3977.18, *p* < 0.001; Table 3), which ranged from 31 to 39.9% with the highest in PGPB+AMF+NPK treatment and the lowest in the control. The zinc content ranged between 2.7 and 4.9 mg 100 g^–1^ (F*_9,20_* = 1842.11, *p* < 0.001; Table 3) and iron ranged between 16.8 and 19.9 mg 100 g^–1^ across treatments, again with the highest in PGPB+AMF+NPK addition, as compared to the lowest in the control.

**TABLE 3:** Nutrient contents of soybean grains as affected by inoculation of plant growth-promoting bacteria (PGPB) and arbuscular mycorrhiza fungi (AMF), with or without the addition of nitrogen (N), phosphorus (P), and potassium (K) fertilizers.

Treatments	Carbohydrate	Protein	Zinc	Iron

	____________%_____________		_________mg 100 g^–1^_________	
Control	20.14 ± 0.02f	31.02 ± 0.16h	2.74 ± 0.03h	16.75 ± 0.03f
PGPB	22.23 ± 0.02e	32.38 ± 0.06f	3.21 ± 0.01g	18.32 ± 0.03e
AMF	22.24 ± 0.02e	32.06 ± 0.06g	3.19 ± 0.02g	18.23 ± 0.02e
PGPB+AMF	22.33 ± 0.02d	32.75 ± 0.06e	3.33 ± 0.03f	18.43 ± 0.02d
PGPB+N	22.30 ± 0.02d	38.65 ± 0.19c	3.46 ± 0.07e	18.49 ± 0.03d
PGPB+PK	22.30 ± 0.02d	32.65 ± 0.10e	4.74 ± 0.02c	19.64 ± 0.08b
PGPB+NPK	22.37 ± 0.02c	38.33 ± 0.10d	4.73 ± 0.02c	19.71 ± 0.02b
PGPB+AMF+N	22.34 ± 0.02cd	39.60 ± 0.10b	3.64 ± 0.03d	18.60 ± 0.01c
PGPB+AMF+PK	22.41 ± 0.01b	38.85 ± 0.04c	4.82 ± 0.03b	19.72 ± 0.08b
PGPB+AMF+NPK	22.67 ± 0.02a	39.92 ± 0.04a	4.92 ± 0.05a	19.90 ± 0.02a

Data (Mean ± SD) within columns with different letters are significantly different (Tukey’s HSD, *p* < 0.001).

### Correlation of soil parameters and soybean performance

**FIGURE 7:**
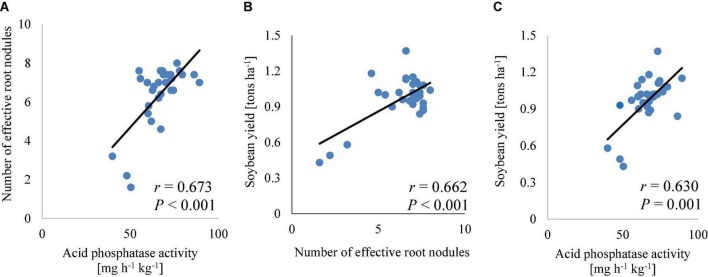
Correlation of the number of effective root nodules and acid phosphatase activity **(A)**, soybean yield and number of effective root nodules **(B)**, or acid phosphatase activity **(C)**.

The soil phosphorus correlated significantly (*p* < 0.05; Table 4) with protein (*r* = 0.46) and carbohydrate (*r* = 0.41) contents in soybean grains. The effective root nodules correlated significantly (*p* < 0.001) with acid phosphatase (*r* = 0.67; Figure 7A) and soybean yield (*r* = 0.66; Figure 7B). Acid phosphatase correlated significantly (*p* < 0.001) with soybean yield (*r* = 0.63; Figure 7C) and carbohydrate (*r* = 0.61; Table 4) contents in the grains. The effective root nodules correlated significantly with carbohydrate (*r* = 0.87, *p* < 0.001), protein (*r* = 0.46, *p* < 0.01), zinc (*r* = 0.59, *p* < 0.001), and iron (*r* = 0.77, *p* < 0.01) contents in soybean grains (Table 4). These correlations indicate higher root nodulation with increased acid phosphatase activity in the rhizosphere of soybean plants, and the influence of symbiotic N_2_ fixation and plant available phosphorus in soil on the nutrient contents of soybean grains. Overall, these results highlight strong relationships between farm management practices, rhizosphere microbial activities, and soybean grain bio-fortification.

**TABLE 4:** Correlation of nutrient value of soybean grains with the number of effective root nodules, acid phosphatase activity in the rhizosphere, and plant available phosphorus in soil.

Nutrient contents	Number of effective nodules	Acid phosphatase activity	Plant available phosphorus in soil
of soybean grains	*r* values	*r* values	*r* values
Carbohydrate	0.87***	0.61***	0.41*
Protein	0.46**	ns	0.46*
Zinc	0.59***	ns	ns
Iron	0.77**	ns	ns

Values are significant at *p < 0.05, **p < 0.01, and ***p < 0.001; ns, not significant.

## Discussion

### Mycorrhization and root nodulation

Although soil N and P did not differ significantly across treatments, increased microbial activities (root nodulation, mycorrhization, and acid phosphatase) in the rhizosphere resulting from the influence of inoculated microorganisms probably increased plant nutrient uptake, which resulted in greater yield (Rawat et al., 2020). The fact that mycorrhiza colonization was not observed in plants that were not inoculated with AMF likely reflects low native AMF presence in soil, while a consistent level of AMF colonization for all inoculated treatments reflects positive plant–AMF symbiosis, especially under low native AMF status. However, the addition of PGPB and NPK fertilizer did not affect the rate of AMF colonization compared to the sole AMF treatment, indicating a strong boost of the inoculated AMF to compensate for low native AMF presence, irrespective of other inputs. Chen et al. (2018) and Powell and Rillig (2018) also described such a low AMF colonization rate as an indication of low soil health status. This could be attributed to intensive and unsustainable farm management practices such as chemical inputs and soil tillage under rotational cropping systems that may affect the soil biota (Wakam et al., 2015; Begoude et al., 2016). The observed high root colonization in AMF inoculated plants demonstrates the capacity of the inoculated mycorrhiza to compete with other rhizosphere microbiota and survive, which is an important characteristic of efficient bio-inoculants in enhancing soil fertility and productivity.

The high root nodulation achieved with inoculation of the PGPB consortium comprising *Bradyrhizobium* suggests successful symbiosis between the inoculated indigenous rhizobia and the soybean roots (Korir et al., 2017; Soumare et al., 2020). Accordingly, improved soybean root nodulation and N_2_ fixation were reported with the inoculation of *Bradyrhizobium* species and fertilizer addition (Ulzen et al., 2016; Leggett et al., 2017; Dabesa and Tana, 2021). Moreover, the high root nodulation in soybean rhizosphere inoculated with PGPB comprising symbiotic *Bradyrhizobium* and non-symbiotic bacteria species is consistent with Jabborova et al. (2021) who reported higher root nodulation and nitrogen-fixing capacity following co-inoculation of *B. japonicum* and *Pseudomonas putida* compared to uninoculated plants. Additionally, Akley et al. (2022) reported a positive residual effect of *Bradyrhizobium* inoculation on soybean root nodulation after three cropping seasons. Nonetheless, root nodulation by soybean plants was not favored by inoculation of mycorrhiza, which is not consistent with other reports on the contribution of mycorrhiza to the N_2_ fixing process (Daniel et al., 2020; Novais et al., 2020). The poor root nodulation in the control can be attributed to a low density of native N_2_ fixing symbiotic rhizobium and low soil phosphorus content (Rosenblueth et al., 2018; Soumare et al., 2020). Although high plant available N in the soil can reduce or inhibit the symbiotic effectiveness of introduced rhizobia strains (Kasper et al., 2019), the addition of N to PGPB-inoculated soybean plants did not reduce root nodulation. However, the present results do not support our hypothesis that co-inoculating plant growth-promoting bacteria and mycorrhiza will enhance soybean root nodulation. This may be due to nutrient deficiencies especially low soil phosphorus as Püschel et al. (2017) reported that the contribution of AMF to N_2_ fixation by rhizobium can be affected by environmental factors and nutrient availability including soil phosphorus.

### Acid phosphatase activity

Soil phosphatase enzymes can be produced by plant roots or rhizosphere microorganisms and they play key roles in catalyzing reactions associated with organic matter decomposition and P cycling, while their quick response to changes in soil management is considered a useful biological indicator (Chodak and Nikliñska, 2012; Rejsek et al., 2012; Behera et al., 2017). The low acid phosphatase activity recorded in the rhizosphere of non-inoculated soybean plants is likely the contributions of soybean roots and the indigenous soil microbial community, while high phosphatase activity in the inoculated plants reflects the additional influence of the inoculated microbes that are involved in mineralizing organic to inorganic P (Rawat et al., 2020), or solubilizing inorganic phosphates (Tchakounté et al., 2020). The high acid phosphatase activity in sole PGPB treatment compared to the control and sole AMF treatments highlights the potential of inoculated plant growth-promoting bacteria. Some microbes in the inoculant biofertilizer used in this study were isolated from the rhizosphere of maize plants in Cameroon, with demonstrated ability to solubilize rock phosphate, fix N_2_, and produce siderophores (Tchakounté et al., 2018, 2020). Therefore, acid phosphatase activity in the rhizosphere can be explored as a possible mode of action of the inoculated microbes to induce nutrient dynamics that may enhance crop productivity. The superior acid phosphatase activity in the soybean rhizosphere inoculated with PGPB comprising symbiotic *Bradyrhizobium* and non-symbiotic bacteria species is in line with Jabborova et al. (2021) study which reported a higher acid phosphatase activity following co-inoculation of *B. japonicum* and *P. putida*. The increase in phosphatase activity following mycorrhiza inoculation could have been due to the production of glycoproteins (e.g., glomalin-related soil proteins) that increased microbial and enzymatic activities (Agnihotri et al., 2022). The observed decrease in acid phosphatase activity in the rhizosphere of PGPB-inoculated soybean plants and amended with NPK is in line with the demonstrated ability of NPK fertilizers to reduce soil microbial functions and acid phosphatase activity as reported by Mndzebele et al. (2020). These results are consistent with the second hypothesis that inoculating plant growth-promoting bacteria and arbuscular mycorrhiza fungi will enhance acid phosphatase activity in the rhizosphere of soybean plants, although their co-inoculation did not produce greater effects.

### Soybean grain yield and nutrient contents

The soybean grain yield in the control plots for this study is within the range of 448–709 kg ha^–1^ that was previously reported by Wendt and Atemkeng (2004), across the first and second planting seasons in the humid forest ecosystem of Cameroon. However, treatment applications in this study increased the soybean grain yield relative to the control and the previously reported yield by Wendt and Atemkeng (2004). Inoculating PGPB and AMF with or without NPK fertilizer significantly increased soybean yield as compared to the control, which supports the third hypothesis that PGPB and AMF will increase soybean grain yield and nutrient contents (Thioub et al., 2017). Ksiȩżak and Bojarszczuk (2022) also reported higher soybean yield following inoculation with *Bradyrhizobium* and fertilizer addition. This increased soybean yield is consistent with the role of inoculated PGPB that comprise some *Bacillus* strains in nutrient solubilization and siderophore production as reported by Tchakounté et al. (2018, 2020). This is supported by Radhakrishnan et al. (2017) who highlighted the role of *Bacillus* strains in mediating crop growth *via* secretion of metabolites, drought tolerance, and protection against pests and diseases. The inoculated microbes in this study likely facilitated the conversion of complex forms of essential nutrients (e.g., P and N) to simple available forms for uptake by plant roots leading to enhanced growth and yield (Kang et al., 2015; Kuan et al., 2016). Furthermore, the secretion of phosphatase and organic acids by *Bacillus* spp. probably facilitated the conversion of inorganic phosphate into plant available phosphate for root uptake (Kang et al., 2014, 2015). Additionally, the inoculated microbes may have released ammonia from nitrogenous organic matter in the soil or their *nifH* genes produced nitrogenase for N_2_ fixation and uptake by plant roots to enhance growth and yield as reported for *Bacillus* spp. (Ding et al., 2005; Hayat et al., 2010; Kuan et al., 2016). The inoculation of mycorrhiza likely enhanced the production of glycoproteins that improved soil quality *via* increased aggregation and carbon sequestration, water-holding capacity, nutrient storage and availability, microbial and enzymatic activities, and production of extracellular polysaccharides, which increased crop performance (Agnihotri et al., 2022). The role of microbial inoculation in the present study is supported by correlations of soybean grain yield with microbial activities in the rhizosphere such as acid phosphatase and root nodulation, which is consistent with Lamptey et al. (2014). The superior performance of soybean plants that were inoculated with the consortium of PGPB comprising *Bradyrhizobium* is supported by Leggett et al. (2017), who reported improved soybean growth, nodulation and N_2_ fixation, and grain yield following inoculation with *Bradyrhizobium*. Meanwhile, the lack of grain yield increase when NPK fertilizers were added to PGPB or AMF indicates that the additional NPK inputs were not enough to cause a significant difference in this study. This finding opens up avenues for further investigation on the appropriate NPK fertilizer amounts to integrate with microbial inoculants.

The higher nutrient content in soybean grains inoculated with PGPB comprising symbiotic *Bradyrhizobium* and non-symbiotic bacteria species is in line with Jabborova et al. (2021) study which reported superior plant nutrients following inoculation of *B. japonicum* and *P. putida* compared to uninoculated plants. Akley et al. (2022) also reported a positive residual effect of *Bradyrhizobium* inoculation on soybean yield after three cropping seasons. The high nutrient contents in soybean grains from plants inoculated with PGPB and AMF, with NPK fertilizer addition in this study indicates soil nutrient enhancement and their effective uptake by plants that eventually accumulated in the grains. Similar results were reported by Sogut (2016), Marro et al. (2020), and Zhang et al. (2020). Beneficial microorganisms may directly enhance nutrient uptake and accumulation or indirectly stimulate other biochemical processes that are involved in the mobilization and deposition of nutrients in the grains (Lamptey et al., 2014; Marro et al., 2020). Overall, the improved nutrient contents in soybean grains following co-inoculation of plant growth-promoting bacteria and AMF with or without NPK fertilizer addition likely results from a combination of increased biochemical processes as reported by Sheteiwy et al. (2021), and supports the concept of integrated soil fertility management (Vanlauwe et al., 2010). These results confirm the positive impact of microbial inoculation and fertilizers on grain yield and protein content of maize (Martins et al., 2017) and soybeans (Marro et al., 2020; Alemayehu and Tana, 2021; Ksiȩżak and Bojarszczuk, 2022). Yasmin et al. (2020) also reported higher nutrient contents (e.g., N, P, K, Ca, Mg) in sweet potato roots following inoculation with *Klebsiella* sp. and N fertilizer addition. Nitrogen is an important component that is responsible for several physiological and biochemical processes in plants, being a structural constituent of chlorophyll molecules, proteins, enzymes, and nucleic acids (Nunes-Nesi et al., 2010). Hence, plants with proper nutrition *via* the application of chemical and biofertilizers likely had higher chlorophyll content with increased photosynthesis and production of photoassimilates, grain filling, and chemical composition, which increased soybean yield and nutritive contents. Ramesh et al. (2014) and Khande et al. (2017) reported that *Bacillus* species substantially influenced the mobilization of zinc and its concentration in soybean, which can be utilized as bio-inoculants for bio-fertilization and bio-fortification. Iron chelation by *Bacillus* spp. *via* siderophore production facilitates iron solubilization from minerals and organic compounds in the rhizosphere by binding Fe^3+^ in complex substances and reducing them to Fe^2+^ for uptake by plants (Walker and Connolly, 2008; Nadeem et al., 2012). These reports support the potential of our locally produced inoculant biofertilizer comprising three *Bacillus* strains with the potential to produce siderophore that likely increased mobilization of zinc for uptake by soybean plants.

## Conclusion

The absence of mycorrhizal colonization in non–AMF-inoculated plants compared to the inoculated ones demonstrates very low content of native AMF strains in the Yaoundé field site in Cameroon, but there was no significant difference between the AMF-inoculated treatments. The successful mycorrhizal colonization of soybean roots for plants inoculated with AMF coupled with the significantly higher soybean yield highlights the potential of AMF inoculation to boost soybean productivity as compared to the control. Sole inoculation of PGPB or AMF-enhanced root nodulation and acid phosphatase activities in the rhizosphere of soybean plants, but their co-inoculation was not significantly different from the sole inoculations. The inoculation of PGPB and AMF increased soybean grain yield and bio-fortification, while NPK fertilizer addition enhanced soybean grain bio-fortification. Overall, these results open up important pathways for further investigation on local strategies for sustainable integrated soil fertility management to boost the productivity of soybean.

## Data availability statement

The original contributions presented in this study are included in the article/Supplementary material, further inquiries can be directed to the corresponding author.

## Author contributions

CN conceived the study, performed data analysis and literature searches, and prepared the first manuscript draft. BT participated in conceiving the study, established the field experiment, performed literature searches, and reviewed the manuscript. MO produced the inoculant formulations of plant growth-promoting bacteria, contributed in establishing the field experiment and literature searches, and reviewed the manuscript. CS participated in conceiving the study and coordinated the establishment and management of the field experiment. RN participated in the field establishment and literature searches and read the manuscript draft. MN participated in literature searches and read the manuscript draft. DA participated in data processing and analysis and literature searches and read the manuscript draft. GT isolated and plant growth-promoting bacteria and coordinated the production of microbial inoculum, and read the manuscript draft. SR coordinated the study and read the manuscript draft. All authors read the draft manuscript and approved the final manuscript.
