# Supplementary material for: Inoculating plant growth-promoting bacteria and arbuscular mycorrhiza fungi modulates rhizosphere acid phosphatase and nodulation activities and enhance the productivity of soybean (Glycine max)
Source: Front Plant Sci. 2022 Sep 26;13:934339. doi: 10.3389/fpls.2022.934339 (PMC9549076; doi:10.3389/fpls.2022.934339)
Supplement: Supplementary file 1 [file Table_1.pdf]

Supplementary data

Table S1. Soybean root nodulation as affected by inoculation of plant growth-promoting bacteria (PGPB) and arbuscular mycorrhiza fungi (AMF), with or without addition of nitrogen (N), phosphorus (P) and potassium (K) fertilizers.

Treatments	Nodulation rating	Volume of nodules/plant (mL)	Nodule dry weight (g)
Control	4.73 ± 1.15b	0.16 ± 0.02c	0.017 ± 0.002b
PGPB	9.80 ± 1.11a	0.34 ± 0.05a	0.037 ± 0.003a
AMF	7.40 ± 0.72a	0.23 ± 0.05b	0.021 ± 0.002b
PGPB+AMF	10.20 ± 0.53a	0.39 ± 0.02a	0.036 ± 0.001a
PGPB+N	10.40 ± 1.22a	0.35 ± 0.01a	0.035 ± 0.002a
PGPB+PK	9.67 ± 1.68a	0.39 ± 0.03a	0.037 ± 0.002a
PGPB+NPK	10.20 ± 1.22a	0.39 ± 0.02a	0.038 ± 0.002a
PGPB+AMF+N	9.80 ± 0.92a	0.34 ± 0.02a	0.036 ± 0.003a
PGPB+AMF+PK	10.33 ± 0.64a	0.37 ± 0.03a	0.035 ± 0.003a
PGPB+AMF+NPK	9.73 ± 1.10a	0.40 ± 0.01a	0.038 ± 0.001a

Data (Mean ± SD) within columns with different letters are significantly different (Tukey's HSD, $P < 0.05$).
